# Correction

**DOI:** 10.1080/19420862.2025.2574103

**Published:** 2025-10-12

**Article title**: A highly selective TCR-mimic antibody reveals unexpected mechanisms of HBV peptide-MHC recognition and previously unknown target biology

**Authors**: Khan, S., Lum, J., Stephenson, H., Kohli, P. B., Mortenson, D., Ramakrishnan, D., Hung, M., Ding, S., Seto, E., Lu, S., Yen, R., Jin, D., Lee, B., Clancy, S., Oakdale, N. S., Novikov, N., Kang, D., Li, R., Pan, D., Dave, R., Lansdon, E., Fletcher, S. P., Garg, A. V., Thomsen, N., & Balsitis, S.

**Journal**: *mAbs*

**DOI**: https://doi.org/10.1080/19420862.2025.2562998

The author has reported that Figure 4 appears incorrect in the published article and has requested that it be updated to display correctly.

**Figure 4:**
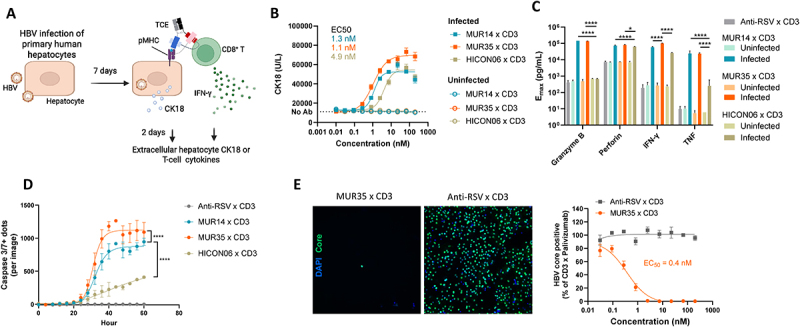

The author has requested that the current versions of Figure 4 be replaced with the updated versions provided below, as they more accurately reflect the original intentions.

